# Risk factors for acute renal impairment in patients with severe acute pancreatitis

**DOI:** 10.1186/cc13386

**Published:** 2014-03-17

**Authors:** M Serpytis, N Scupakova, J Sabliauskas, A Sileikis, J Sipylaite, K Strupas

**Affiliations:** 1 Vilnius University, Vilnius, Lithuania

## Introduction

The aim of this study was to clarify the risk factors for acute renal impairment (ARI) in patients with severe acute pancreatitis (SAP).

## Methods

We conducted a retrospective observational case-control study. The data of all patients admitted to the ICU at a university hospital with the diagnosis of SAP from January 2008 to December 2012 were abstracted from the hospital database. ARI was defined according to RIFLE criteria. Patients with any signs of renal impairment (serum creatinine value ≥1.5-fold from the estimated baseline) on admission or history of kidney disease were excluded. A control group was composed of randomly selected patients with SAP who did not develop ARI.

## Results

Of 145 patients, 24 patients who developed ARI at any time during hospitalization (ARI group) were contrasted with 24 patients without ARI (control group). The patients were older in the ARI group: age 57 ± 13 versus 49 ± 16, *P *= 0.046. Although none of the patients in the ARI group had creatinine values ≥1.5-fold from the estimated baseline, the creatinine values on admission were higher in this group: 100 ± 38 versus 68 ± 16, *P *= 0.001. The severity of pancreatitis was similar. On admission, the APACHE II and SOFA scores were higher in the ARI group (12.7 ± 3.7 vs. 8.6 ± 3.4, *P *= 0.001 and 5.6 ± 3.4 vs. 2.8 ± 1.9, *P *= 0.002, respectively). The patients in the ARI group had higher intraabdominal pressure and SOFA respiratory score on admission and after 72 hours (*P *< 0.01). Although on admission the cardiovascular SOFA score was similar in both groups, it increased significantly after 72 hours in the ARI group from 1.1 ± 1.7 to 2.1 ± 1.8, *P *= 0.012 and became higher compared with the control group: 2.1 ± 1.8 versus 0.6 ± 1.2, *P *= 0.001. Lactate was higher in the ARI group on admission and after 72 hours (*P *< 0.05). The percentage of patients requiring vasopressors over 72 hours was greater in the ARI group: 66.7% versus 29.2%, *P *= 0.01. Positive fluid balance after 72 hours in ICU was higher in the ARI group: 8,594 ± 7,044 ml versus 4,192 ± 4,467 ml, *P *= 0.004; however, the infused volume of crystalloids did not differ between groups. Central venous pressure was higher on admission (*P *= 0.036) and after 72 hours (*P *= 0.001) in the ARI group. Multivariable logistic regression analysis revealed that development of ARI was independently associated with cardiovascular SOFA score after 72 hours: odds ratio = 1.475, 95% CI 1.049 to 2.073, *P *= 0.025.

## Conclusion

The development of ARI in SAP was associated with hemodynamic instability, whereas excessive volume expansion does not prevent ARI.

